# Plasma 25-Hydroxyvitamin D Level and VDR Gene Single Nucleotide Polymorphism rs2228570 Influence on COVID-19 Susceptibility among the Kazakh Ethnic Group—A Pilot Study

**DOI:** 10.3390/nu15071781

**Published:** 2023-04-06

**Authors:** Valeriya V. Protas, Gayane P. Pogossyan, Konstantin G. Li, Assel G. Zhumina, Anar K. Bisseneva, Dinara N. Shaikina

**Affiliations:** 1 Department of Botany, Karaganda Buketov University, Karaganda 100028, Kazakhstan; 2 Biotechnology and Eco-Monitoring Research Park, Karaganda Buketov University, Karaganda 100028, Kazakhstan; 3 Department of Biology, Non-Profit Limited Company “Manash Kozybayev North Kazakhstan University”, Petropavlovsk 150000, Kazakhstan

**Keywords:** vitamin D, 25(OH)D, COVID-19, susceptibility, genetic polymorphism rs2228570, vitamin D receptor (VDR) gene

## Abstract

Low plasma levels of the vitamin D metabolite 25-hydroxyvitamin D [25(OH)D] and the vitamin D receptor (VDR) gene single nucleotide polymorphisms (SNPs) have been associated with the body’s susceptibility to infectious diseases, including COVID-19. In this pilot retrospective study, representatives of the Kazakh population (central Kazakhstan) were divided into groups based on the test for IgM and IgG for coronavirus infection. We compared the 25(OH)D plasma levels and concluded that the COVID-19-positive group values (25.17 ng/mL ± 16.65) were statistically lower (*p* = 0.0114) compared to the COVID-19-negative ones (35.58 ng/mL ± 20.67). There was no association between age, gender and 25(OH)D concentration within the groups (*p* > 0.05). The genotyping of rs2228570 was performed using a TaqMan Real-Time PCR assay. Allele C predominated among the COVID-19-negative participants and significantly reduced the likelihood of coronavirus infection (*p* < 0.0001; OR = 0.0804; 95% CI 0.02357–0.2798). There were no statistically significant differences in the frequencies of the A, G and T alleles in the studied groups (*p* > 0.05). The GG genotype of rs2228570 was associated with a 4.131-fold increased likelihood of COVID-19 infection (*p* = 0.0288; χ^2^ = 5.364; OR = 4.131; 95% CI 1.223–13.71). Comprehensive studies are required to determine whether low 25(OH)D plasma concentrations and genetic background represent a risk factor for COVID-19 infection.

## 1. Introduction

COVID-19 caused by the SARS-CoV-2 coronavirus was first reported in China at the end of 2019. The pandemic has spread to all countries in the world [1]. Multiple cases of infection continue to be recorded even now [2]. Clinical manifestations of coronavirus infection can vary from the mildest form (asymptomatic) to serious conditions (acute respiratory distress syndrome (ARDS), pneumonia, mortality) [3]. Therefore, urgent tasks for global scientific research are to find ways and means to control the incidence of and mortality from coronavirus infection, as well as to study the molecular genetic basis of the body’s predisposition to this disease [4,5,6].

Vitamin D is a member of the secosteroid hormones class and is known for its role in phosphorus and calcium metabolism regulation [7,8,9]. The nutrient enters the human body in two ways: exogenously, i.e., with food (including nutritionals), and endogenously, when the synthesis occurs directly in the skin with the participation of ultraviolet rays [5,10]. Both forms of vitamin D require further activation, which occurs in two stages. Primary hydroxylation occurs in the liver, where 25-hydroxyvitamin D (25(OH)D or calcidiol) is formed [7,11,12]. 25(OH)D is excreted from the body within 2–3 weeks, so its plasma concentration is a frequently used criterion for determining vitamin D status and is measured for evaluation [11,12,13]. The second stage of hydroxylation occurs in the kidneys, where the vitamin D biologically active systemic circulating form—calcitriol—is formed (1.25-dihydroxyvitamin D, 1α,25(OH)_2_D_3_) [12].

Vitamin D production and activation in the body mainly depend on ultraviolet exposure, age, nutrition and ethnicity. The 7-dehydrocholesterol reductase (DHCR7) and CYP2R1 gene polymorphisms, vitamin D binding protein and vitamin D receptor (VDR) are important factors as well [7].

The active form of vitamin D is known for both its role in osteomineral metabolism and pleiotropic effects, including immune response regulation [4,14,15]. Calcitriol has an effect on innate and adaptive immunity. It suppresses the production of cytokines and, thus, reduces the invading pathogen load. On the other hand, calcitriol reduces the excessive activation of the adaptive immune system, helping the body to balance the immune response and adequately respond to infection [11,16].

Vitamin D deficiency is a major public health problem worldwide [14]. A cross-sectional survey by Gromova et al. revealed 25(OH)D deficiency in six regions of Kazakhstan [17]. Insufficient vitamin D levels are related to reduced autoimmunity and increased susceptibility to infections [14]. Vitamin D preventive medication has been reported to decrease the risk of respiratory tract infections [18] and influenza, especially in winter [19]. There is also evidence that optimal serum vitamin D levels are associated with a reduced likelihood of viral infections, including the human immunodeficiency virus, fever and hepatitis B and C [5]. The pandemic, which began in 2019, has sparked a new research wave aimed at studying the protective functions of vitamin D, due to COVID-19 being an infectious respiratory disease accompanied by an extreme inflammatory response [5,10].

Currently, a sufficient number of publications have already examined vitamin D’s association with the incidence of coronavirus infection. A number of researchers have found that low vitamin D levels increase the likelihood of SARS-CoV-2 infection [14,20,21,22,23], whereas other authors reported no effect of vitamin D status on susceptibility to COVID-19 [24,25]. Several studies reported the presence of a correlation only for Asian countries’ populations, but none for those of European countries [14,24]. Such discrepancies can be explained by differences in study design, as well as ethnicity, age and clinical and other differences in the studied samples. There are no peer-reviewed publications on similar studies in Kazakhstan.

Active vitamin D is also a transcription factor. Its genomic effects are mediated by the intracellular vitamin D receptor. VDR belongs to the superfamily of nuclear steroid hormone receptors [26] and is expressed in almost all body tissues, including immune cells [27,28]. Activated by the ligand, VDR is involved in the control of several thousand genes’ transcription, in particular ACE2, known as the receptor for SARS-CoV-2 [29,30]. The activity and structure of the VDR protein depends on the VDR gene, its expression and polymorphisms [6,31,32].

The VDR gene is localized on chromosome 12q13.11 and is polymorphic [6]. Its single nucleotide polymorphisms (SNPs) can affect VDR products (mRNA and/or proteins), which ultimately leads to impaired vitamin D immune regulatory functions [33]. One of the best-studied VDR gene SNPs is the four-allelic rs2228570 [34]. Currently, there is enough research on the effect of rs2228570 on predisposition to various diseases, such as atopic bronchial asthma [27], acute viral bronchiolitis [35], tuberculosis [36,37], dengue virus infection [38], respiratory syncytial virus-related disease [39] and others. There are publications studying the potential impact of genetic variations in the VDR gene (including rs2228570) on COVID-19 susceptibility and severity, but their number is small and the results differ significantly [33,40,41,42].

Therefore, the present study’s purpose is to evaluate the potential effects of vitamin D and rs2228570 on susceptibility to COVID-19 among Kazakh ethnic group representatives.

## 2. Materials and Methods

### 2.1. Study Subjects

The study included 119 Kazakh ethnic group representatives of both sexes over 18 years old, residing in the city of Karaganda and the Karaganda region (Kazakhstan Republic). The selection of Kazakh participants for the study was based on a questionnaire. All participants had not been vaccinated against COVID-19 in the last 12–18 months and did not take vitamin D supplements. Full biometric, laboratory and genetic data of the study participants are provided in the Supplementary Materials (Table S1). The study was conducted in accordance with the recommendations of the Helsinki Declaration and approved by the Local Bioethics Committee Non-commercial joint-stock company “Karaganda Medical University” (protocol No. 2 dated 11 October 2022). All participants gave written informed consent.

### 2.2. Plasma IgG and IgM for SARS-CoV-2 and Plasma 25(OH)D Assays

Blood samples were taken from voluntary participants from October 2022 to November 2022. Blood sampling was carried out in two sterile evacuated tubes with K3-EDTA, and samples were subsequently centrifuged. The plasma was placed into polypropylene tubes. The samples were tested for M and G immunoglobulins for coronavirus infection using the SARS-CoV-2-IgG-ELISA-BEST and SARS-CoV-2-IgM-ELISA-BEST test systems (Vector-Best, Novosibirsk, Russia). 25(OH)D plasma concentration was measured using a “25OH Vitamin D Total ELISA” (Demeditec Diagnostics GmbH, Kiel, Germany). All analyses were performed in accordance with the instructions.

### 2.3. The rs2228570 of VDR Gene Genotyping

Total DNA was extracted from whole blood using an RIBO-prep kit (Amplisens, Moscow, Russia). The analysis was carried out in accordance with the instructions. DNA concentration and purity were measured on a DS-11 spectrophotometer (DeNovix Inc., Wilmington, DE, USA). rs2228570 genotyping was tested by using a TaqMan Real-Time PCR assay. The final volume of the PCR reaction mixture was 25 μL, which included 50 ng DNA, 10 pmol of each primer (Lumiprobe, Moscow, Russia), 15 pmol TaqMan probes (Lumiprobe, Moscow, Russia), Taq polymerase, dNTP and the buffer (GeneLab, Astana, Kazakhstan). Amplification was performed in a DTlite real-time PCR cycler (DNA-Technology, Moscow, Russia) using the Real-Time_PCR v.7.9 software (DNA-Technology, Moscow, Russia). The standard cycling conditions for TaqMan Real-Time PCR were initial enzyme activation for 3 min at 94 °C; 40 cycles: 15 s at 94 °C (denaturation) and 62 °C for 30 s (annealing/elongation). The primer sequences (Lumiprobe, Moscow, Russia) were as follows: VDR gene rs2228570 forward primer (5′-TCCACACACCCCACAGATCC-3′), VDR gene rs2228570 reverse primer (5′-GTGGGTGGCACCAAGGATG-3′).

### 2.4. Statistical Analysis

Continuous data are presented as mean ± standard deviation (SD) and were tested for normal distribution using the Shapiro–Wilk test. The categorical variables are described as frequencies and percentages. The comparison of continuous variables between the two groups was conducted using the Mann–Whitney test followed by power analysis. Multiple comparisons were examined using the Kruskal–Wallis statistical test, followed by Dunn’s multiple comparison test. Spearman’s rank correlation was used to determine the continuous data. Fisher’s exact test and the χ^2^ test were used to compare categorical data. Tests for deviations from the Hardy–Weinberg equilibrium (HWE) were conducted using a chi-square distribution. The odds ratios (OR) and 95% confidence intervals are reported; two-sided *p* < 0.05 was considered statistically significant. GraphPad Prism 8.0 software (Graph-Pad Software, San Diego, CA, USA) was used for performing statistical analysis. Power was computed using a post hoc test via G*Power software (Version 3.1.9.4, Heinrich Heine Universität, Düsseldorf, Germany).

## 3. Results

### 3.1. Study Flow

This study included 119 participants of the Kazakh ethnic group from the Karaganda city and region. 

All participants’ blood samples were analyzed to determine the level of M and G immunoglobulins for coronavirus infection. It is known that antibody titers to COVID-19 begin to simultaneously increase in the period from 7 to 28 days after infection [43,44,45]. In addition, the level of IgM decreases after 50 days [46], and it is hardly detected 3 months after the onset of symptoms [45,47]. IgG persists after the seventh week [48] and declines between 4 and 7 months [46,47]. 

Therefore, the presence of IgM or IgG in the plasma above the threshold (>1 ng/mL) indicates current infection with SARS-CoV-2 or past infection in the interval from a week to six months prior to the analysis. Consequently, according to the ELISA analysis results, all participants were conditionally divided into two groups:

Group 1: COVID-19-positive (p-COVID-19). One third of p-COVID-19 participants (*n* = 27, 30.7%) had IgM > 1 ng/mL, i.e., had been recently infected. Most of the p-COVID-19 group (*n* = 61, 69.3%) had IgG > 1 ng/mL with a negative result for IgM (<1 ng/mL), which indicates they had been infected and recovered. The 25(OH)D concentration in the recently infected part of the group was 24.98 ± 18.04 ng/mL (*n* = 27). This indicator in the second part of the group was 25.25 ± 16.16 ng/mL (*n* = 61). The mean value comparison according to the nonparametric Mann–Whitney test showed no statistically significant differences (*p* = 0.7720) in the 25(OH)D levels between the parts of the p-COVID-19 group, divided by the time of infection. The results confirm the possibility of considering them as a single p-COVID-19 participant group. 

Group 2: COVID-19-negative (no-COVID-19). This group was negative for all antibody types for COVID-19 (IgM < 1 ng/mL; IgG < 1 ng/mL).

Summary data on participants are shown in Table 1. A slight smoker excess was found in the p-COVID-19 group (*n* = 21; 23.9%) compared to the no-COVID-19 group (*n* = 5; 16.1%; χ^2^ = 0.8032; *p* = 0.3701). In both groups, smoking was more common among men (*n* = 15; 71.4% and *n* = 4; 80.0%). For women, this factor was 28.6% in the p-COVID-19 and 20.0% in the no-COVID-19 group. 

### 3.2. Plasma 25(OH)D Concentration

**Figure 1:**
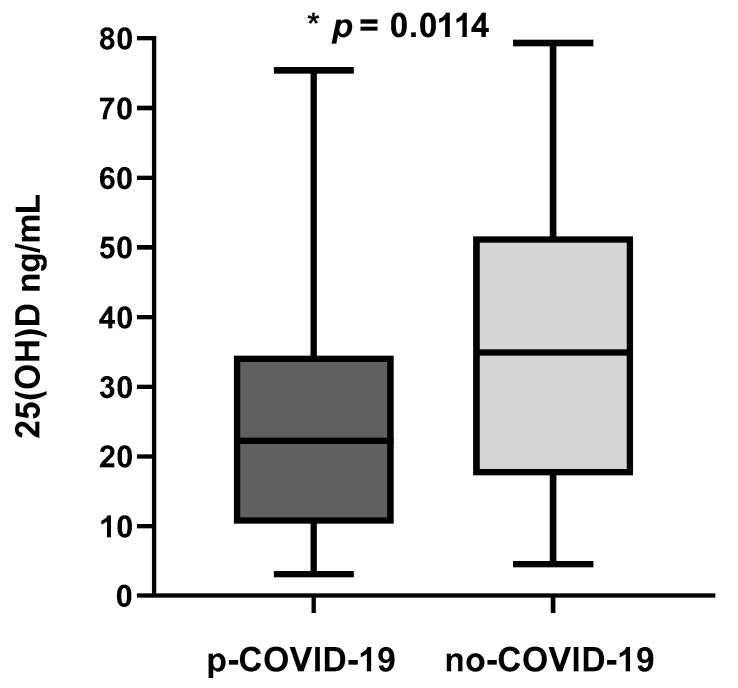
Plasma 25(OH)D levels in blood samples of COVID-19-positive and COVID-19-negative subjects; * *p* = 0.0114.

We compared the 25(OH)D plasma concentrations between p-COVID-19 and no-COVID-19 participants. This indicator in the p-COVID-19 group (25.17 ± 16.65 ng/mL; *n* = 88) was statistically lower (*p* = 0.0114) compared to the no-COVID-19 group (35.58 ± 20.67 ng/mL; *n* = 31) (Figure 1). 

We assessed the deficiency of 25(OH)D (>20 ng/mL) in the groups as well. Deficiencies occurred almost 1.5 times more often among COVID-19-positive participants than among COVID-19-negative ones (*n* = 38; 43.2% vs. *n* = 9; 29.0%; *p* = 0.2026). Notably, acute deficiency (>10 ng/mL) was identified in 22.7% of the p-COVID-19 group, which was 3.5 times more common than in the no-COVID-19 group (*n* = 2; 6.5%; OR = 4.265; 95% CI = 1.078–19.29; *p* = 0.0447).

**Figure 2:**
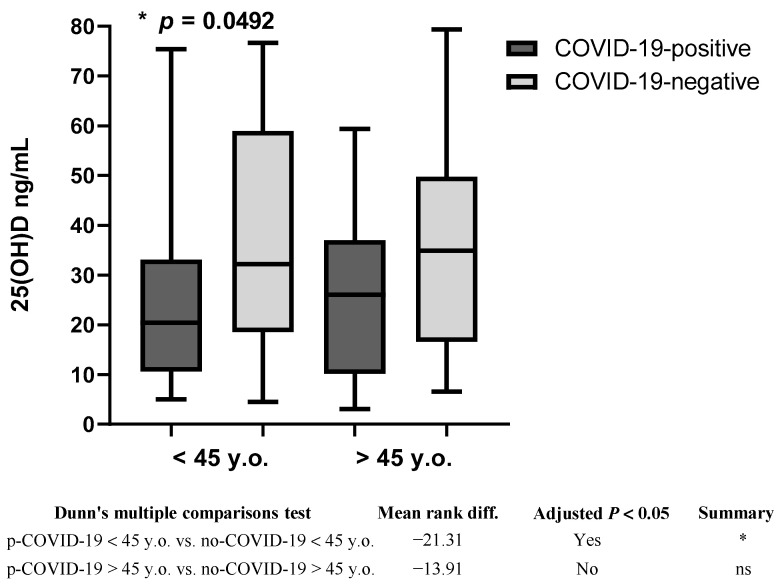
Plasma 25(OH)D levels among COVID-19 positive and COVID-19 negative participants in different age groups; * *p* = 0.0492, ns = not significant; y.o. = years old.

Since the median age of participants was close to 45, the younger participants were specified as the “younger’’ group and those over 45 years as the “older” ones. There were no significant differences (*p* = 0.04724) in 25(OH)D concentrations between the younger and older groups in general (27.20 ± 18.89 ng/mL; *n* = 68 vs. 28.78 ± 17.58 ng/mL; *n* = 51) (Figure S1). However, 25(OH)D concentrations in the younger p-COVID-19 group participants (24.05 ± 17.13 ng/mL; *n* = 50) were significantly lower (*p* = 0.0492) than in the younger no-COVID-19 participants (35.94 ± 21.21 ng/mL; *n* = 18). No statistically significant differences were detected in the older groups (*p* = 0.4191) (Figure 2).

The 25(OH)D levels in men and women were comparable in general (21.84 ± 33.72 ng/mL vs. 23.84 ± 32.01 ng/mL; *p* = 0.9989). This trend also persisted when comparing with groups (*p* = 0.0636; Figure S2). 

The participants’ diet was not studied. The body mass index (BMI; kg/m^2^) in both groups was similar (26.44 ± 4.479 kg/m^2^ for p-COVID-19 and 26.22 ± 4.098 kg/m^2^ for no-COVID-19), and these values indicate a sufficient amount of nutrients in the diet. 

**Figure 3:**
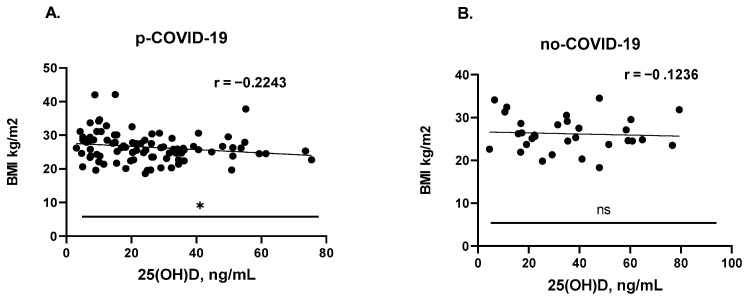
Relationship between plasma 25(OH)D levels and BMI among p-COVID-19 (**A**) and no COVID-19 (**B**) participants; * *p* = 0.357, ns = not significant; r = correlation coefficient.

As shown in Figure 3A, there was a statistically significant inverse correlation between BMI and 25(OH)D plasma levels in the p-COVID-19 group (r = −0.2243; *p* = 0.0357). A similar, but slightly lower, weak inverse relationship (r = −0.1236; *p* = 0.5076) between these parameters was found in the no-COVID-19 group (Figure 3B). Evidently, the reason lies in the smaller sample size of the no-COVID-19 group (*n* = 31 vs. *n* = 88). The results obtained are consistent with the conclusions presented in a number of other studies [49,50,51]. 

We compared 25(OH)D concentrations among smokers and non-smokers within the study groups and found no significant differences (*p* > 0.05). However, the remarkable fact is that 25(OH)D levels in both groups were lower among participants who smoked. The same trend persisted in the study of gender-related differences. The lowest 25(OH)D values were recorded among male smokers in the p-COVID-19 group (20.674 ± 10.84 ng/mL; *n* = 15), while the highest values were recorded among uninfected non-smoker males (42.47 ± 10.29 ng/mL; *n* = 9).

### 3.3. 25(OH)D Concentration and rs2228570 

No significant associations (*p* = 0.0823) between certain rs2228570 alleles and 25(OH)D plasma concentration were found in the groups (Figures S3 and S4). 25(OH)D plasma concentrations were in the range of 20.68–27.02 ng/mL in the p-COVID-19 group, regardless of rs2228570 genetic variants. In addition, this indicator in the no-COVID-19 group was within the “normal” range (31.68–44.10 ng/mL). It should be noted that the 25(OH)D mean values corresponded to the norm in both groups in the presence of the homozygous AA genotype.

### 3.4. rs2228570 and COVID-19

To identify the potential role of the VDR gene SNP rs2228570 in susceptibility to COVID-19, we analyzed the allele and genotype frequencies within the p-COVID-19 and no-COVID-19 groups. Both groups were in Hardy–Weinberg equilibrium (*p* = 0.9994 and *p* = 0.5099, respectively). The results of the rs2228570 genotyping are shown in Table 2.

According to Table 2, the C allele frequencies significantly differed between the studied groups (*p* < 0.0001). It significantly predominated among the no-COVID-19 participants (*n* = 11; 17.74%) compared to the p-COVID-19 participants (*n* = 3; 1.70%). 

Despite the revealed rare frequency of the C allele in the Kazakh population, it is significantly related to the reduced likelihood of SARS-CoV-2 infection (χ^2^ = 21.30; OR = 0.0804; 95% CI 0.02357–0.2798; *p* < 0.0001) and can be considered as a potential security marker. This is also confirmed by the fact that similar differences in frequency were found for genotypes that include the C allele (AC and CG). The frequency of the AC genotype among the no-COVID-19 group was 12.90%, while in the p-COVD-19 group, this genetic variation was detected only in 1.14% of cases (χ^2^ = 7.886; OR = 0.07759; 95% CI 0.006289–0.5156; *p* = 0.0161). The CG genotype was also significantly more frequently detected in the no-COVID-19 group (*n* = 5; 16.13%; χ^2^ = 7.950; OR = 0.1209; 95% CI 0.02351–0.6340; *p* = 0.0128) compared with the p-COVID-19 group (*n* = 2; 2.27%). The CC genotype was extremely rare, but the only participant (*n* = 1; 3.23%) was in the no-COVID-19 group.

We observed that the homozygous GG genotype was significantly more common in the p-COVID-19 group (*n* = 27; 30.68%; χ^2^ = 5.364; OR = 4.131; 95% CI 1.223–13.71; *p* = 0.0288) in comparison with the no-COVID-19 group (*n* = 3; 9.68%).

No statistically significant differences were determined in the A, G and T allele frequencies of the studied groups (*p* > 0.05). We should note that the TT and AT genotypes, which are rare in the Kazakh population (*n* = 2.27% and *n* = 9.09%, respectively), were exclusively detected in the p-COVID-19 group. 

## 4. Discussion

The functions of vitamin D as a pluripotent hormone are extremely diverse. Currently, its participation in the implementation of immune responses, anti-inflammatory and antimicrobial activity has become beyond dispute [5,14,52].

In this retrospective pilot study, we analyzed the 25(OH)D plasma concentration in COVID-19-positive and COVID-19-negative representatives of the Kazakh ethnic group to determine the potential impact of vitamin D on susceptibility to coronavirus infection. The main result was the detection of lower vitamin D plasma levels (measured as 25(OH)D) in the p-COVID-19 group compared to the no-COVID-19 group.

These results are consistent with a number of studies reporting that SARS-CoV-2-positive patients have significantly lower 25(OH)D levels compared to negative patients [9,14,21,53,54]. Similar outcomes were demonstrated in the Gallelli et al. study, where participants were divided into three groups depending on the duration of infection. The lowest levels of 25(OH)D were found in patients with acute infection (9.63 ± 8.70 ng/mL), slightly higher in recovered ones (11.52 ± 4.90 ng/mL) and highest in non-infected ones (15.96 ± 5.99 ng/mL) [55]. A large observational population study conducted by Israel et al. demonstrated that low levels of 25(OH)D are associated with a higher risk of SARS-CoV-2 infection, even after adjusting for confounding factors such as geographical region, socioeconomic status, ethnicity and comorbidities [56]. The meta-analysis conducted by Mukherjee and colleagues also revealed that a reduction in vitamin D levels is associated with an elevated risk of COVID-19 infection. The authors considered seasonal UV exposure as the main cause of this correlation [57].

A retrospective study by Kaufman et al. reported that positive tests for SARS-CoV-2 were more common in “deficient” 25(OH)D (<20 ng/mL) patients compared to “adequate” (30–34 ng/mL) ones [21]. In this study, deficiency was found 1.5 times more often in the COVID-19 group; however, the difference was not statistically significant. It should be noted that acute vitamin D deficiency was associated with a 4.265-fold increased likelihood of SARS-CoV-2 infection.

Gender differences may influence vitamin D levels [58]. In some studies, lower 25(OH)D concentrations are more commonly associated with females [17,58], whereas the others are associated with males [59,60]. In our research, men and women had comparable levels of 25(OH)D within study groups, showing no relationship between vitamin D concentration and gender. Similar results were reported in a study by Zhumina et al., conducted in the same Kazakhstan region, comparing 25(OH)D concentrations among healthy volunteers separated by sex [61].

The influence of genetic factors on vitamin D is still being studied. Collective data from several studies have shown the potential importance of the CG, DHCR1, CYP2R1, CYP24A1 and VDR genes in the hormone metabolism [62,63]. The most-studied single nucleotide polymorphisms of the VDR gene are rs1544410, rs731236, rs7975232 and rs2228570 [63]. In the current study, we examined the relationship between rs2228570 genotypes or alleles and the participants’ 25(OH)D plasma concentration. No correlation was found in the study groups. Data from other authors on this issue are also extremely contradictory as well. For example, studies in China and Russia have demonstrated that lower 25(OH)D levels are associated with the ff genotype rs2228570 (FokI T > C) [64,65], while deficiency in the Syrian population was associated with the FF genotype [66]. Three more research teams (namely, two from India and one from Greece) showed no relationship between genetic variations in rs2228570 and 25(OH)D concentrations [63,67,68]. Despite the fact that no correlation between the 25(OH)D level and rs2228570 was found, it becomes promising to conduct studies with a larger sample. 

A meta-analysis by Laplana et al., which revealed the association of the rs2228570 VDR gene with viral infections, led to the study of a potential relationship between this SNP and susceptibility to COVID-19. The authors stated that the T allele and its homozygous variant (TT genotype) are risk factors for respiratory syncytial virus (RSV) infection [69]. RSV belongs to the enveloped viruses group including SARS-CoV-2. Based on this, VDR gene SNPs are considered potentially significant in the study of COVID-19 [70]. Currently, there are several publications considering rs2228570 as a factor influencing the susceptibility, clinical symptoms and severity of COVID-19, but their number is small [33,40,41,42]. Abdollahzadeh et al. revealed the association of SNP rs2228570 (FokI) with fever and arterial hypertension in severe/critical patients [33]. There are two opposing points of view concerning the connection between the coronavirus infection severity and the rs2228570 genetic variations. Apaydin et al. found an association between the heterozygous FokI (TC) genotype and disease severity [42], while Kotur et al. showed that rs2228570 (A > G) variants are not related to a higher risk of severe COVID-19 [41]. The only study on the effect of certain VDR gene SNPs on susceptibility to COVID-19 was carried out among an Iranian population by Jafarpoor et al. The authors evaluated four SNPs of the VDR gene, including rs2228570 (C > T). The CT (heterozygous) genotype has been significantly associated with increased likelihood of COVID-19 compared to CC (homozygous reference standard). In addition, the T allele is also associated with a high susceptibility to SARS-CoV-2 infection [40].

Our study shows that the C allele of rs2228570 significantly reduces the likelihood of COVID-19 (OR = 0.0804; 95% CI 0.02357–0.2798; *p* < 0.0001). The AC and CG genotypes are also associated with a lower chance of infection. The CC genotype did not show significant protective properties due to the extremely rare detection; however, the sole participant with the CC genotype was found among the uninfected group. The data obtained for the C allele are generally consistent with Jafarpoor et al. [40]. The effect of A, G and T alleles on susceptibility to coronavirus infection has not been defined. However, two of the three genotypes with the T allele (TT and AT) were found only in the p-COVID-19 participants. Due to the rare frequency of T alleles in the Kazakh population, it cannot be argued that it is associated with increased likelihood of COVID-19; however, this can be assumed indirectly.

The frequency of genotypes AA, AG and TG in the p-COVID-19 and no-COVID-19 groups was comparable. The Kazakh population did not have any CT genotypes. However, the rs2228570 GG genotype was associated with a 4.131-fold increased likelihood of COVID-19 infection.

We understand that our research is not free from limitations. The first reason for this is the retrospective study design. According to the results of ELISA for IgM and IgG for coronavirus infection without a PCR test, the 25(OH)D plasma concentration during infection becomes impossible accurately determine. The second one is the small sample size and unequal gender distribution. With regard to the third reason, the participants’ diets were not studied and there was no access to clinical data.

However, this is the first study conducted in Kazakhstan that revealed significant differences in the 25(OH)D levels between participants who were recently infected or recovered and COVID-19 negative ones. It is also one of the few studies evaluating the association of single-nucleotide polymorphisms in the VDR gene, in particular rs2228570, with susceptibility to COVID-19.

## 5. Conclusions

This pilot study conducted in Central Kazakhstan demonstrates statistically lower plasma concentrations of 25(OH)D in COVID-19-positive Kazakhs compared to COVID-19-negative ones. There was an association between rs2228570 of the VDR gene and COVID-19. The C allele of rs2228570 was associated with reduced likelihood of susceptibility to COVID-19, while the GG genotype increased these odds. Further studies on specific groups with a larger sample size and complete data in the Kazakh population are required.

## Figures and Tables

**Table 1:** The characteristics of the study groups.

	p-COVID-19	no-COVID-19	*p* Value
Total (*n*)	88	31	
Age (years; mean ± SD)	43 ± 14.38	41 ± 15.24	NS
Sex (M/F)	27/61	12/19	
BMI (mean ± SD)	26.44 ± 4.479	26.22 ± 4.098	NS
Tobacco use (M/F)	21 (15/6)	5 (4/1)	
IgM (ng/mL; mean ± SD)	1.691 ± 3.008	0.399 ± 0.181	****
IgG (ng/mL; mean ± SD)	7.086 ± 3.881	0.3191 ± 0.2282	****
25(OH)D (ng/mL; mean ± SD)	25.17 ± 16.65	35.58 ± 20.67	*

* = *p* < 0.05, **** = *p* < 0.0001, NS = not significant.

**Table 2:** rs2228570 allele and genotype frequencies in p-COVID-19 and no-COVID-19 groups.

rs2228570	p-COVID-19 (*n*)	no-COVID-19 (*n*)	OR (CI 95%)	χ^2^	*p*-Value ^#^
Genotypes	*n* = 88 (Freq.)	*n* = 31(Freq.)			
AA	9 (10.23%)	2 (6.45%)	1.652 (0.3850–7.963)	0.3895	0.7260
CC	NA	1 (3.23%)	0.1149 (0.004558–2.895)	2.863	0.2605
GG	27 (30.68%)	3 (9.68%)	4.131 (1.223–13.71)	5.364	0.0288 *
TT	2 (2.27%)	NA	1.821 (0.08506–38.97)	0.7166	ref (1.00)
AC	1 (1.14%)	4 (12.90%)	0.07759 (0.006289–0.5156)	7.886	0.0161 *
AG	27 (30.68%)	12 (38.71%)	0.7008 (0.3047–1.566)	0.6706	0.5051
AT	8 (9.09%)	NA	6.652 (0.3728–118.7)	3.021	0.1096
CG	2 (2.27%)	5 (16.13%)	0.1209 (0.02351–0.6340)	7.950	0.0128 *
TG	12 (13.64%)	4 (12.90%)	1.066 (0.3389–3.226)	0.01059	ref (1.00)
Alleles	*n* = 176 (Freq.)	*n* = 62 (Freq.)			
A	54 (30.68%)	20 (32.26%)	0.9295 (0.497–1.724)	0.05317	0.8736
C	3 (1.70%)	11 (17.74%)	0.0804 (0.02357–0.2798)	21.30	<0.0001 ****
G	95 (53.98%)	27 (43.55%)	1.520 (0.8606–2.755)	1.996	0.1843
T	24 (13.64%)	4 (6.45%)	2.289 (0.7916–6.330)	2.280	0.1702

**^# ^***p*-values were calculated using Fisher’s exact test. * = *p* < 0.05, **** = *p* < 0.0001, NA = not available.

## Data Availability

Dataset is available on request.

## Supplementary Materials

The following supporting information can be downloaded at: https://www.mdpi.com/article/10.3390/nu15071781/s1, Table S1: Characteristics of study participants; Figure S1: Plasma 25(OH)D levels in blood samples of COVID-19-positive and COVID-19-negative participants separated by age; Figure S2: Plasma 25(OH)D levels in blood samples of COVID-19-positive and COVID-19-negative participants separated by sex; Figure S3: Plasma 25(OH)D levels in COVID-19-positive subjects’ blood samples and rs2228570 alleles; Figure S4: Plasma 25(OH)D levels in COVID-19-negative subjects’ blood samples and rs2228570 alleles; ns = not significant.
