# Supplementary material for: Plasma 25-Hydroxyvitamin D Level and VDR Gene Single Nucleotide Polymorphism rs2228570 Influence on COVID-19 Susceptibility among the Kazakh Ethnic Group—A Pilot Study
Source: Nutrients. 2023 Apr 6;15(7):1781. doi: 10.3390/nu15071781 (PMC10097393; doi:10.3390/nu15071781)
Supplement: Supplementary file 1 [file nutrients-15-01781-s001.zip › nutrients-2295717-supplementary.pdf]

Supplementary Materials

Supplementary Table S1. Characteristics of study participants (n = 119)

No.	Sex	Age	BMI[a]	Tobacco Use	IgG ng/mL	IgM ng/mL	25(OH)D ng/mL	Test Date	rs2228570 genotype
1	M	21	20.1	Yes	0.16	5.4	18.295	17.10.22	AA
2	F	20	28.6	No	0.13	0.34	16.922	17.10.22	AC
3	F	53	26.8	No	8.2	0.54	17.569	17.10.22	AA
4	M	30	22.7	No	3.09	0.64	20.704	17.10.22	AA
5	M	49	22.1	Yes	8.5	8.5	9.974	17.10.22	AC
6	F	22	23.5	No	0.22	0.45	76.622	17.10.22	AC
7	F	39	25.1	Yes	0.41	0.31	21.317	19.10.22	AG
8	F	32	29.5	No	2.12	0.57	49.812	19.10.22	AA
9	M	69	28.4	No	9.69	7.49	7.227	19.10.22	AG
10	M	36	23.1	Yes	8.1	0.78	30.817	19.10.22	GG
11	F	36	20.6	Yes	1.2	9.8	5.021	19.10.22	GG
12	F	38	25.3	No	0.95	2.89	73.59	21.10.22	AG
13	F	59	32.4	No	0.19	0.31	11.373	21.10.22	AC
14	M	53	25.6	Yes	7.98	5.73	24.419	21.10.22	AG
15	F	65	20.3	No	8.96	1.67	29.102	21.10.22	GT
16	F	46	19.8	No	0.18	0.4	25.422	21.10.22	GG
17	F	48	26.7	No	8.43	1.22	39.137	21.10.22	GT
18	F	59	24.8	No	7.91	1.74	23.75	21.10.22	GG
19	F	55	27.5	No	1.27	0.45	26.146	21.10.22	GC
20	M	58	26.4	Yes	0.23	0.3	17.303	21.10.22	AG
21	F	56	33.7	Yes	9.44	1.16	7.306	21.10.22	AT
22	M	74	34.1	Yes	0.32	0.46	6.537	21.10.22	AG
23	F	74	24.6	No	11.73	7.49	4.683	21.10.22	GT
24	M	35	31.1	No	10.14	21.17	9.377	21.10.22	AG
25	F	19	21.3	No	0.21	0.47	29.223	21.10.22	GG
26	F	35	23.7	No	0.62	0.4	19.116	21.10.22	AG
27	F	23	21.9	No	0.97	0.56	16.807	21.10.22	AG
28	M	48	24.5	No	0.73	0.79	35.435	24.10.22	CC
29	M	18	25.2	Yes	10.88	0.86	15.641	24.10.22	AA
30	F	69	37.8	No	11.52	1.04	55.223	24.10.22	AG
31	F	60	26.5	No	10.61	0.76	29.888	24.10.22	GT
32	F	77	20.3	No	3.243	0.77	32.327	24.10.22	AG
33	F	56	30.5	No	0.121	0.18	34.933	24.10.22	GT
34	F	33	24.5	No	8.98	0.78	61.419	24.10.22	GG
35	F	50	26.7	No	6.8	0.51	48.064	24.10.22	AA
36	F	51	24.6	No	0.14	0.66	59.122	25.10.22	AG
37	F	46	27.5	No	0.18	0.62	39.81	25.10.22	AG
38	M	23	24.8	Yes	0.3	0.47	64.782	25.10.22	AA
39	M	74	30.6	No	11	0.6	40.651	25.10.22	GG
40	F	30	25.7	Yes	3.42	0.56	40.651	25.10.22	GG
41	F	54	27.9	Yes	10.07	0.28	54.895	25.10.22	AG
42	F	27	42.0	No	4.51	0.63	8.777	25.10.22	GG
43	F	25	21.4	No	8.45	1.27	34.495	25.10.22	GT
44	M	30	26.1	No	3.17	0.28	14.832	25.10.22	AG
45	F	49	32.5	No	10.24	0.59	20.188	25.10.22	AG
46	M	20	28.6	Yes	11.14	0.63	5.101	25.10.22	AT

47	F	38	25.9	No	0.7	0.47	22.296	25.10.22	GC
48	F	50	29.4	No	9.06	0.69	7.250	25.10.22	AT
49	F	64	31.1	No	9.47	0.3	4.260	25.10.22	GT
50	F	45	27.1	No	0.2	0.23	58.440	25.10.22	GT
51	F	48	25.8	No	3.9	0.59	7.375	25.10.22	GT
52	F	48	23.5	No	8.53	0.29	26.737	25.10.22	AG
53	F	41	29.5	No	0.18	0.17	60.312	25.10.22	GC
54	F	57	25.0	No	7.12	0.36	44.743	25.10.22	GT
55	F	54	34.6	No	4.31	0.34	10.210	27.10.26	TT
56	F	27	27.9	Yes	7.58	0.28	6.222	27.10.26	GG
57	F	32	22.6	No	0.3	0.95	4.509	27.10.26	GC
58	M	61	28.3	No	0.16	0.2	31.486	27.10.26	GT
59	F	51	22.4	No	9.57	0.3	19.789	27.10.26	GT
60	M	68	26.2	Yes	0.19	0.23	15.858	27.10.26	AC
61	F	49	19.7	No	3.45	0.38	50.882	27.10.26	AA
62	F	38	26.2	No	6.3	0.33	53.75	27.10.26	GG
63	F	20	28.5	No	1.95	8.93	12.552	27.10.26	AG
64	F	37	23.8	No	6.78	0.8	10.283	27.10.26	GG
65	M	38	30.1	Yes	8.45	0.27	35.981	27.10.26	AT
66	F	45	24.9	No	5.54	1.08	20.397	27.10.26	AT
67	F	45	27.5	No	2.7	0.27	21.385	27.10.26	AG
68	F	36	30.6	No	1.46	0.23	28.634	27.10.26	TT
69	M	54	42.1	No	4.39	0.59	15.024	31.10.22	GG
70	F	33	22.7	No	12.76	0.34	34.518	31.10.22	AT
71	F	38	25.5	Yes	13.11	0.96	21.574	31.10.22	GT
72	F	48	23.7	No	0.26	0.18	51.559	31.10.22	GT
73	M	18	18.3	No	0.55	0.32	47.883	31.10.22	GC
74	M	18	24.7	Yes	11.64	0.33	33.697	31.10.22	GG
75	F	42	29.4	No	11.23	0.61	5.087	31.10.22	GG
76	F	42	26.3	No	3.04	0.28	16.922	31.10.22	AT
77	F	38	23.8	No	3.23	0.44	51.236	31.10.22	AG
78	F	36	27.9	No	2.28	0.19	14.994	31.10.22	AT
79	M	48	26.4	No	1.15	0.21	18.207	03.11.22	GT
80	F	42	25.6	No	0.17	0.28	22.491	03.11.22	AG
81	F	42	32.8	No	1.61	0.57	12.351	03.11.22	GG
82	F	47	31.8	No	0.26	0.35	79.334	03.11.22	GC
83	M	34	28.6	Yes	1.76	0.59	8.357	03.11.22	AG
84	F	44	31.1	No	5.19	0.51	10.71	03.11.22	AG
85	F	60	34.2	No	12.8	0.51	9.945	03.11.22	GG
86	F	59	28.5	No	13.34	0.32	24.098	03.11.22	GG
87	F	27	23.4	No	4..83	0.59	7.268	03.11.22	GG
88	F	23	19.6	No	6.71	0.28	9.027	03.11.22	AG
89	F	42	25.8	No	5.99	0.35	32.895	03.11.22	GG
90	F	39	29.1	No	7.75	0.43	32.513	03.11.22	GG
91	M	31	24.5	No	0.87	0.51	60.854	03.11.22	AG
92	F	18	21.4	No	5.65	1.05	11.504	03.11.22	GG
93	F	31	25.6	No	2.71	0.95	36.208	03.11.22	GC
94	M	35	25.3	No	0.15	0.42	38.551	03.11.22	GG
95	M	53	30.4	Yes	6.66	2.12	26.345	03.11.22	GG
96	M	45	29.1	No	0.19	0.49	35.201	03.11.22	AG
97	F	48	24.7	No	8.4	0.46	31.68	03.11.22	AG
98	F	45	27.5	No	11.22	0.51	22.88	03.11.22	AG

99	M	75	26.3	No	12.08	3.73	51.202	03.11.22	GG
100	F	65	25.9	No	11.46	1.63	34.214	03.11.22	AG
101	F	21	30.1	No	11.38	5.59	15.488	04.11.22	AG
102	M	58	24.3	Yes	6.5	0.46	9.335	04.11.22	GG
103	M	54	34.5	No	0.17	0.23	47.872	04.11.22	AA
104	M	23	18.6	No	1.41	2.18	24.288	04.11.22	AG
105	M	34	23.8	Yes	2.83	0.42	7.504	04.11.22	AG
106	F	43	27.6	No	14.7	0.48	14.291	04.11.22	GG
107	M	41	22.7	No	5.36	0.6	75.44	04.11.22	AG
108	M	22	19.5	No	2.03	1.6	24.905	04.11.22	AG
109	F	43	26.3	No	5.66	1.6	29.107	04.11.22	AG
110	F	48	22.4	No	12.8	0.89	36.255	04.11.22	GG
111	M	47	24.5	No	12.64	2.39	59.341	04.11.22	AA
112	M	33	21.7	No	13.7	0.97	15.449	04.11.22	AG
113	F	38	27.8	No	13.09	2.44	20.366	04.11.22	GT
114	M	47	26.2	No	10.96	7.43	3.089	04.11.22	GG
115	M	58	24.7	Yes	2.92	0.39	35.448	04.11.22	AG
116	F	35	23.5	No	3.9	0.29	26.301	04.11.22	GG
117	M	18	20.3	No	0.26	0.28	41.008	04.11.22	AG
118	F	38	31.3	No	0.33	0.34	10.583	04.11.22	AG
119	F	60	19.7	No	4.64	0.32	25.942	04.11.22	AA

[a] BMI. body mass index;

Supplementary Figure S1. Plasma 25(OH)D levels in blood samples of COVID-19-positive and COVID-19-negative participants separated by age; ns = not significant; y.o.. years old.

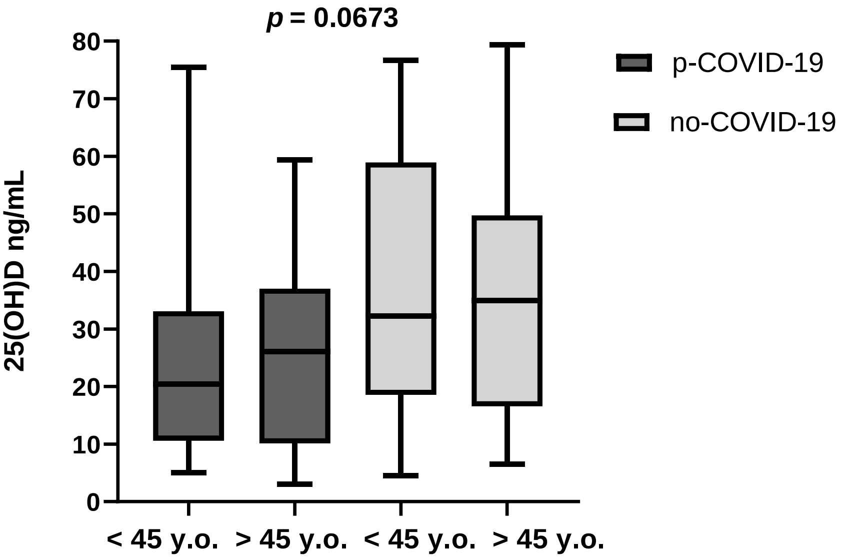

Dunn's multiple comparisons test	Mean rank diff.	Adjusted *P* < 0.05	Summary
p-COVID-19 < 45 y.o. vs. p-COVID-19 > 45 y.o.	−6.712	No	ns
no-COVID-19 < 45 y.o. vs. no-COVID-19 > 45 y.o.	0.6944	No	ns

Supplementary Figure S2. Plasma 25(OH)D levels in blood samples of COVID-19-positive and COVID-19-negative participants separated by sex; ns = not significant

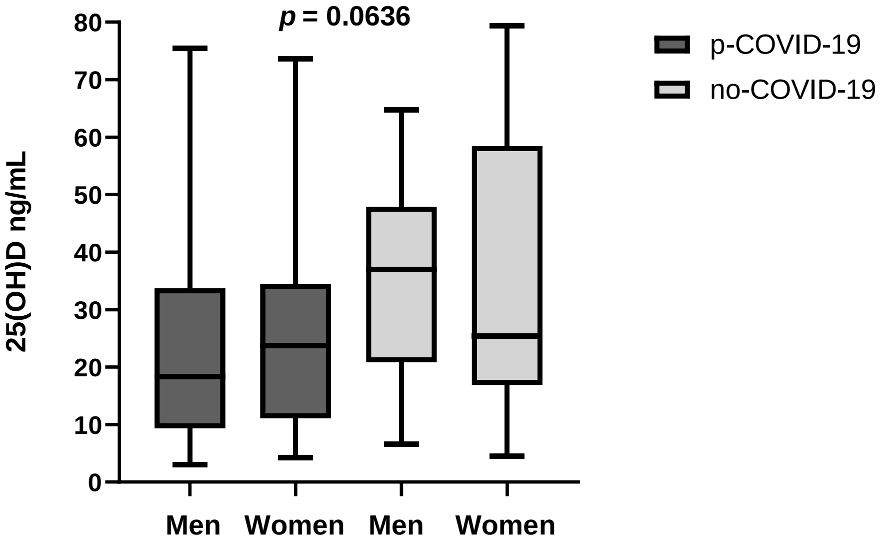

Dunn's multiple comparisons test	Mean rank diff.	Adjusted *P* < 0.05	Summary
p-COVID-19 men vs. p-COVID-19 women	−5.344	No	ns
no-COVID-19 men vs. no-COVID-19 women	8.996	No	ns

Supplementary Figure S3. Plasma 25(OH)D levels in COVID-19-positive subjects' blood samples and rs2228570 alleles; ns = not significant

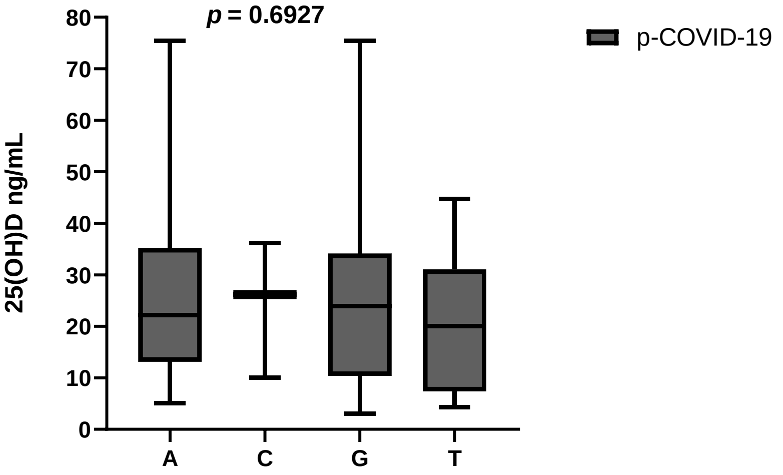

Dunn's multiple comparisons test	Mean rank diff.	Adjusted *P* < 0.05	Summary
A vs. C	−2.894	ref (1.00)	ns
A vs. G	2.824	ref (1.00)	ns
A vs. T	12.05	ref (1.00)	ns
C vs. T	5.718	ref (1.00)	ns
G vs. T	14.94	ref (1.00)	ns
C vs. G	9.221	ref (1.00)	ns

Supplementary Figure S4. Plasma 25(OH)D levels in COVID-19-negative subjects' blood samples and rs2228570 alleles; ns = not significant

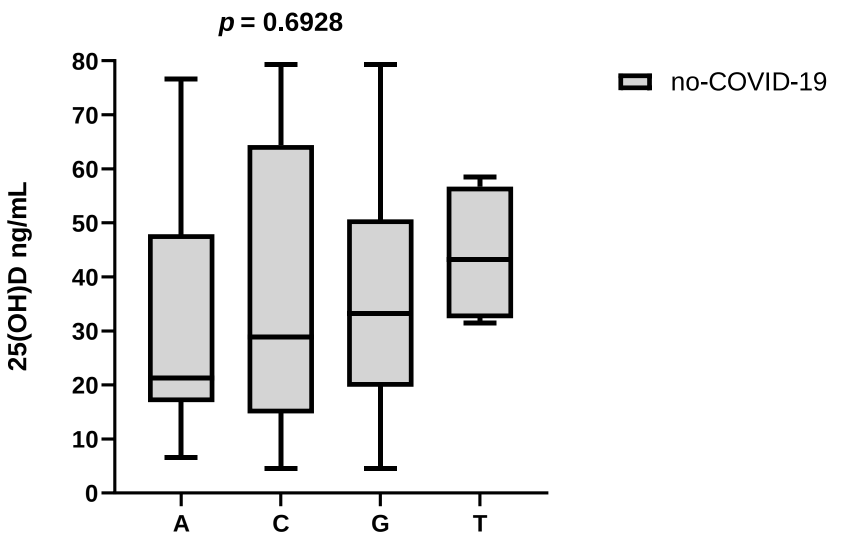

Dunn's multiple comparisons test	Mean rank diff.	Adjusted $P < 0.05$	Summary
A vs. C	−2.682	ref (1.00)	ns
A vs. G	−3.361	ref (1.00)	ns
A vs. T	−10.63	ref (1.00)	ns
C vs. T	−0.6792	ref (1.00)	ns
G vs. T	−7.950	ref (1.00)	ns
C vs. G	−7.271	ref (1.00)	ns

Power analysis of the data shown in Figure 1

t tests - Means: Wilcoxon-Mann-Whitney test (two groups)

**Options:**	A.R.E. method		
**Analysis:**	Post hoc: Compute achieved power		
**Input:**	Tail(s)	=	One
	Parent distribution	=	Normal
	Effect size d	=	0.5521576
	α err prob	=	0.05
	Sample size group 1	=	88
	Sample size group 2	=	31
**Output:**	Noncentrality parameter δ	=	2.5834345
	Critical t	=	1.6586177
	Df	=	111.6366
	Power (1-β err prob)	=	0.8219635
